# Interim Estimates of Vaccine Effectiveness of BNT162b2 and mRNA-1273 COVID-19 Vaccines in Preventing SARS-CoV-2 Infection Among Health Care Personnel, First Responders, and Other Essential and Frontline Workers — Eight U.S. Locations, December 2020–March 2021

**DOI:** 10.15585/mmwr.mm7013e3

**Published:** 2021-04-02

**Authors:** Mark G. Thompson, Jefferey L. Burgess, Allison L. Naleway, Harmony L. Tyner, Sarang K. Yoon, Jennifer Meece, Lauren E.W. Olsho, Alberto J. Caban-Martinez, Ashley Fowlkes, Karen Lutrick, Jennifer L. Kuntz, Kayan Dunnigan, Marilyn J. Odean, Kurt T. Hegmann, Elisha Stefanski, Laura J. Edwards, Natasha Schaefer-Solle, Lauren Grant, Katherine Ellingson, Holly C. Groom, Tnelda Zunie, Matthew S. Thiese, Lynn Ivacic, Meredith G. Wesley, Julie Mayo Lamberte, Xiaoxiao Sun, Michael E. Smith, Andrew L. Phillips, Kimberly D. Groover, Young M. Yoo, Joe Gerald, Rachel T. Brown, Meghan K. Herring, Gregory Joseph, Shawn Beitel, Tyler C. Morrill, Josephine Mak, Patrick Rivers, Katherine M. Harris, Danielle R. Hunt, Melissa L. Arvay, Preeta Kutty, Alicia M. Fry, Manjusha Gaglani

**Affiliations:** ^1^CDC COVID-19 Response Team; ^2^Mel and Enid Zuckerman College of Public Health, University of Arizona, Tucson, Arizona; ^3^Kaiser Permanente Northwest Center for Health Research, Portland, Oregon; ^4^St. Luke’s, Duluth, Minnesota; ^5^University of Utah, Salt Lake City, Utah; ^6^Marshfield Clinic Research Laboratory, Marshfield, Wisconsin; ^7^Abt Associates, Inc., Atlanta, Georgia; ^8^Leonard M. Miller School of Medicine, University of Miami, Florida; ^9^Baylor Scott & White Health, Temple, Texas; ^10^Whiteside Institute for Clinical Research, St. Luke’s, Duluth, Minnesota; ^11^Texas A&M University College of Medicine, College Station, Texas.

Messenger RNA (mRNA) BNT162b2 (Pfizer-BioNTech) and mRNA-1273 (Moderna) COVID-19 vaccines have been shown to be effective in preventing symptomatic COVID-19 in randomized placebo-controlled Phase III trials (*1*,*2*); however, the benefits of these vaccines for preventing asymptomatic and symptomatic SARS-CoV-2 (the virus that causes COVID-19) infection, particularly when administered in real-world conditions, is less well understood. Using prospective cohorts of health care personnel, first responders, and other essential and frontline workers* in eight U.S. locations during December 14, 2020–March 13, 2021, CDC routinely tested for SARS-CoV-2 infections every week regardless of symptom status and at the onset of symptoms consistent with COVID-19–associated illness. Among 3,950 participants with no previous laboratory documentation of SARS-CoV-2 infection, 2,479 (62.8%) received both recommended mRNA doses and 477 (12.1%) received only one dose of mRNA vaccine.^†^ Among unvaccinated participants, 1.38 SARS-CoV-2 infections were confirmed by reverse transcription–polymerase chain reaction (RT-PCR) per 1,000 person-days.^§^ In contrast, among fully immunized (≥14 days after second dose) persons, 0.04 infections per 1,000 person-days were reported, and among partially immunized (≥14 days after first dose and before second dose) persons, 0.19 infections per 1,000 person-days were reported. Estimated mRNA vaccine effectiveness for prevention of infection, adjusted for study site, was 90% for full immunization and 80% for partial immunization. These findings indicate that authorized mRNA COVID-19 vaccines are effective for preventing SARS-CoV-2 infection, regardless of symptom status, among working-age adults in real-world conditions. COVID-19 vaccination is recommended for all eligible persons.

HEROES-RECOVER^¶^ is a network of longitudinal cohorts in eight locations (Phoenix, Tucson, and other areas in Arizona; Miami, Florida; Duluth, Minnesota; Portland, Oregon; Temple, Texas; and Salt Lake City, Utah) that share a common protocol and methods.** Enrollment in this longitudinal study started in July 2020 and included health care personnel, first responders, and other essential and frontline workers who provided written consent. The current vaccine effectiveness analytic study period began on the first day of vaccine administration at study sites (December 14–18, 2020) and ended March 13, 2021. Active surveillance for symptoms consistent with COVID-19–associated illness (defined as fever, chills, cough, shortness of breath, sore throat, diarrhea, muscle aches, or loss of smell or taste) occurred through weekly text messages, e-mails, and direct participant or medical record reports. Participants self-collected a midturbinate nasal swab weekly, regardless of COVID-19–associated illness symptom status and collected an additional nasal swab and saliva specimen at the onset of COVID-19–associated illness. Specimens shipped on cold packs were tested by RT-PCR assay at Marshfield Clinic Laboratory (Marshfield, Wisconsin) to determine SARS-CoV-2 infections (PCR-confirmed infection). Receipt of COVID-19 vaccines was documented by multiple methods: by self-report in electronic surveys, by telephone interviews, and through direct upload of vaccine card images at all sites; records were also extracted from electronic medical records at the Minnesota, Oregon, Texas, and Utah sites. Among 5,077 participants, those with laboratory documentation of SARS-CoV-2 infection before enrollment starting in July 2020 (608) or identified as part of longitudinal surveillance up until the first day of vaccine administration (240) were excluded. Another 279 were excluded because of low participation (i.e., failed to complete surveillance for ≥20% of study weeks and did not contribute COVID-19–associated illness specimens). Overall, 3,950 participants in the vaccine effectiveness analytic sample were analyzed.

Hazard ratios were estimated by the Andersen-Gill extension of the Cox proportional hazards model, which accounted for time-varying vaccination status. Hazard ratios of unvaccinated person-days to partial immunization person-days (≥14 days after first dose and before second dose) and to full immunization person-days (≥14 days after second dose) were calculated separately. The 13 person-days between vaccine administration and partial or full immunization were considered excluded at-risk person-time because immunity was considered to be indeterminate. Unadjusted vaccine effectiveness was calculated as 100% × (1−hazard ratio). An adjusted vaccine effectiveness model included study site as a covariate. All analyses were conducted with SAS (version 9.4; SAS Institute). This activity was reviewed by CDC and was conducted consistent with applicable federal law and CDC policy.^††^

Approximately one half of the participants (52.6%) were from the Arizona study sites (Table 1). Participants included physicians and other clinical leads (primary health care personnel) (21.1%), nurses and other allied health care personnel (33.8%), first responders (21.6%), and other essential and frontline workers (23.5%). The majority of participants were female (62.1%), aged 18–49 years (71.9%), White (86.3%), and non-Hispanic (82.9%) and had no chronic medical conditions (68.9%). Over the 13-week study period, adherence to weekly surveillance reporting and specimen collection was high (median = 100%; interquartile range = 82%–100%).

**TABLE 1:** Characteristics of health care personnel, first responders, and other essential and frontline workers with reverse transcription–polymerase chain reaction (RT-PCR)–confirmed SARS-CoV-2 infections and percentage receiving one or more doses of a messenger RNA (mRNA) COVID-19 vaccine — eight U.S. locations, December 14, 2020–March 13, 2021

Characteristic	No. (column %) of participants	SARS-CoV-2 infection		Unvaccinated	Vaccinated with ≥1 dose*	
		No. (row %)	p-value^†^	No. (row %)	No. (row %)	p-value^†^
**Total**	**3,950 (100)**	**205 (5.2)**	**—**	**989 (25.0)**	**2,961 (75.0)**	**—**
**Cohort location**						
Phoenix, Arizona	555 (14.1)	39 (7.0^§^)	<0.001	147 (26.5)	408 (73.5)	<0.001
Tucson, Arizona	1,199 (30.4)	79 (6.6^§^)		325 (27.1)	874 (72.9)	
Other, Arizona	320 (8.1)	16 (5.0^§^)		88 (27.5)	232 (72.5)	
Miami, Florida	221 (5.6)	19 (8.6^§^)		118 (53.4)	103 (46.6^¶^)	
Duluth, Minnesota	448 (11.3)	12 (2.7)		47 (10.5)	401 (89.5^¶^)	
Portland, Oregon	468 (11.8)	4 (0.9)		61 (13.0)	407 (87.0^¶^)	
Temple, Texas	289 (7.3)	18 (6.2^§^)		71 (24.6)	218 (75.4)	
Salt Lake City, Utah	450 (11.4)	18 (4.0)		132 (29.3)	318 (70.7)	
**Sex**						
Female**	2,453 (62.1)	109 (4.4)	0.007	529 (21.6)	1,924 (78.4)	<0.001
Male	1,497 (37.9)	96 (6.4)		460 (30.7)	1,037 (69.3)	
**Age group, yrs**						
18–49	2,839 (71.9)	146 (5.1)	0.83	735 (25.9)	2,104 (74.1)	0.48
≥50	1,111 (28.1)	59 (5.3)		254 (22.9)	857 (77.1)	
**Race**						
White	3,408 (86.3)	178 (5.2)	0.92	814 (23.9)	2,594 (76.1)	<0.001
Other	542 (13.7)	27 (5.0)		175 (32.3)	367 (67.7)	
**Ethnicity**						
Hispanic/Latinx	674 (17.1)	57 (8.5)	<0.001	236 (35.0)	438 (65.0)	<0.001
Other	3,276 (82.9)	148 (4.5)		753 (23.0)	2,523 (77.0)	
**Occupation^††^**						
Primary health care personnel	835 (21.1)	16 (1.9)	<0.001	65 (7.8)	770 (92.2)	<0.001
Other allied health care personnel	1,335 (33.8)	67 (5.0)		242 (18.1)	1,093 (81.9)	
First responder	852 (21.6)	75 (8.8)		308 (36.2)	544 (63.8)	
Other essential and frontline worker	928 (23.5)	47 (5.1)		374 (40.3)	554 (59.7)	
**Chronic condition**						
None^§§^	2,723 (68.9)	141 (5.2)	0.92	711 (26.1)	2,012 (73.9)	0.11
≥1	1,227 (31.1)	64 (5.2)		278 (22.7)	949 (77.3)	

* Total vaccinated includes 477 participants who received one mRNA vaccine dose, 2,479 who received two mRNA vaccine doses, and five who received a single dose of the Janssen COVID-19 vaccine (Johnson & Johnson); these five participants contribute unvaccinated person-days until their vaccination date and then no longer contribute to the analysis.

^†^ P-values (comparing the percentage of SARS-CoV-2 infections by sociodemographic and health categories and comparing the percentage vaccinated by these categories) calculated using Pearson's chi-square test (cells with ≥5 observations) or Fisher's exact test (cells with <5 observations).

^§^ Sites identified had statistically higher percentages of participants with RT-PCR-confirmed SARS-CoV-2 infections than the other sites (chi-square = 31.0, p-value <0.001).

^¶^ The Minnesota and Oregon sites had the statistically highest percentage vaccinated with at least one vaccine dose. Florida had the lowest (chi-square = 62.1, p-value <0.001).

** 10 participants were missing biologic sex and were imputed as the more common category (female).

^††^ Occupational categories: primary health care personnel (physicians, physician assistants, nurse practitioners, and dentists), other allied health care personnel (nurses, therapists, technicians, medical assistants, orderlies, and all other persons providing clinical support in inpatient or outpatient settings), first responders (firefighters, law enforcement, corrections, and emergency medical technicians), other essential and frontline workers (workers in hospitality, delivery, and retail; teachers; and all other occupations that require contact within 3 feet of the public, customers, or coworkers as a routine part of their job).

^§§^ 133 participants who did not respond to the self-report question were imputed as “none.”

Most (75.0%) of the participants received one or more doses of vaccine during the study period (Table 1); 477 (12.1%) received their first dose and had not received their second dose by the end of the study period, and 2,479 (62.8%) received both recommended mRNA vaccine doses. Most (60.5%) were vaccinated with their first dose during December 14–31, 2020. Both mRNA vaccine products were administered to participants in all locations but differed in the timing of their availability; 62.7% of vaccinated participants received Pfizer-BioNTech vaccine and 29.6% received Moderna vaccine. The remaining mRNA vaccines (7.7%) are pending product verification. Receipt of at least one vaccine dose was significantly higher among participants who were female, White, non-Hispanic, health care personnel, or living in Minnesota or Oregon; vaccine coverage was lowest in Florida (Table 1).

SARS-CoV-2 infection was diagnosed by RT-PCR in 205 (5.2%) participants; PCR-confirmed infection was significantly higher among participants who were male, Hispanic, first responders, or living in Arizona, Florida, and Texas (Table 1). The majority of PCR-confirmed infections were identified by weekly specimens (58.0%), whereas 42.0% were identified from specimens collected at the onset of COVID-19–associated illness. Nonetheless, the majority (87.3%) of PCR-confirmed infections were associated with symptoms consistent with COVID-19–associated illness. The remaining PCR-confirmed infections were associated with other symptoms not part of the COVID-19–associated illness definition (e.g., headache, fatigue, and rhinorrhea) (2.0%) or no symptoms (10.7%). Only 22.9% of PCR-confirmed infections were medically attended, including two hospitalizations; no deaths occurred.

During the 116,657 person-days when participants were unvaccinated, 161 PCR-confirmed infections were identified (incidence rate = 1.38/1,000 person-days). During the 13 days after first-dose or second-dose vaccination when immune status was considered indeterminate (67,483 person-days), 33 PCR-confirmed infections were identified and excluded from the outcome. Two sources of partially immunized person-days were reported. Five PCR-confirmed infections were reported during 15,868 person-days ≥14 days after their first dose among those who did not receive their second dose during the study period; three PCR-confirmed infections were reported during 25,988 person-days ≥14 days after the first dose and through receipt of the second dose. Taken together, this represents eight PCR-confirmed infections that occurred during 41,856 person-days with partial immunization (≥14 days after first dose and before second dose; incidence rate = 0.19/1,000 person-days). Three PCR-confirmed infections occurred during 78,902 person-days with full immunization (≥14 days after second dose; incidence rate = 0.04/1,000 person-days). Estimated adjusted vaccine effectiveness of full immunization was 90% (95% confidence interval [CI] = 68%–97%); vaccine effectiveness of partial immunization was 80% (95% CI = 59%–90%) (Table 2). In sensitivity analyses, inclusion of other covariates (sex, age, ethnicity, and occupation) were entered individually in the vaccine effectiveness model; the change in vaccine effectiveness point estimates were <3%.

**TABLE 2:** Person-days, SARS-CoV-2 infections, and vaccine effectiveness among health care personnel, first responders, and other essential and frontline workers, by messenger RNA immunization status — eight U.S. locations, December 14, 2020–March 13, 2021

COVID-19 immunization status	Person-days	SARS-CoV-2 infections		Unadjusted vaccine effectiveness*	Adjusted vaccine effectiveness*^,†^
		No.	Incidence rate per 1,000 person-days	% (95% CI)	% (95% CI)
**Unvaccinated**	116,657	161	1.38	N/A	N/A
**Partially immunized**	41,856	8	0.19	82 (62–91)	80 (59–90)
≥14 days after receiving first dose only^§^	15,868	5	0.32		
≥14 days after first dose through receipt of second dose	25,988	3	0.12		
**Fully immunized**					
≥14 days after second dose	78,902	3	0.04	91 (73–97)	90 (68–97)

**Abbreviations:** CI = confidence interval; N/A = not applicable.

* Vaccine effectiveness was estimated using a Cox proportional hazards model accounting for time-varying immunization status.

^†^ Hazard ratio is adjusted for study site.

^§^ Participants received first dose but had not received second dose by the end of the study period.

## Discussion

Prospective cohorts of health care personnel, first responders, and other essential and frontline workers over 13 weeks in eight U.S. locations confirmed that authorized mRNA COVID-19 vaccines (Pfizer-BioNTech’s BNT162b2 and Moderna’s mRNA-1273) are highly effective in real-world conditions. Vaccine effectiveness of full immunization with two doses of mRNA vaccines was 90% (95% CI = 68%–97%) against RT-PCR–confirmed SARS-CoV-2 infection. These findings are consistent with those from the mRNA vaccines’ Phase III trials (*1*,*2*) and recent observational studies of the mRNA vaccine effectiveness against severe COVID-19 (*3*). The findings complement and expand upon these preceding reports by demonstrating that the vaccines can also reduce the risk for infection regardless of COVID-19–associated illness symptom status (*4*,*5*). Reducing the risk for transmissible infection, which can occur among persons with asymptomatic infection or among persons several days before symptoms onset (*6*), is especially important among health care personnel, first responders, and other essential and frontline workers given their potential to transmit the virus through frequent close contact with patients and the public.

Partial immunization (≥14 days after first dose but before second dose) provided preventive benefits with vaccine effectiveness of 80%. This finding is similar to an analysis of Phase III trial results (*1*,*2*,*7*) and two other recent estimates of vaccine effectiveness for partial immunization with Pfizer-BioNTech vaccine among health care personnel, including a vaccine effectiveness (≥21 days after first dose) of 72% (95% CI = 58%–86%) against PCR-confirmed infection identified by routine testing in the United Kingdom (*4*) and a vaccine effectiveness (>14 days after first dose) of 60% (95% CI = 38%–74%) against PCR-confirmed infection identified by records review in Israel (*5*). This finding is also consistent with early descriptive findings of SARS-CoV-2 employee and clinical testing results by mRNA vaccination status in the United States (*8*,*9*).

The findings in this report are subject to at least three limitations. First, vaccine effectiveness point estimates should be interpreted with caution given the moderately wide CIs attributable in part to the limited number of postimmunization PCR-confirmed infections observed. Second, this also precluded making product-specific vaccine effectiveness estimates and limited the ability to adjust for potential confounders; however, effects were largely unchanged when study site was included in an adjusted vaccine effectiveness model and when adjusted for sex, age, ethnicity, and occupation separately in sensitivity analyses. Finally, self-collection of specimens and delays in shipments could reduce sensitivity of virus detection by PCR (*10*); if this disproportionately affected those who received the vaccine (e.g., because of possible vaccine attenuation of virus shedding), vaccine effectiveness would be overestimated.

The scientific rigor of these findings is enhanced by its prospective design and the participants’ very high adherence to weekly specimen collection. As the study progresses, viruses will be genetically characterized to examine the viral features of breakthrough infections. Given that there is uncertainty related to the number of days required to develop immunity postvaccination (*3*–*5*,*7*), future research examining vaccine effectiveness at different intervals is warranted.

These interim vaccine effectiveness findings for both Pfizer-BioNTech’s and Moderna’s mRNA vaccines in real-world conditions complement and expand upon the vaccine effectiveness estimates from other recent studies (*3*–*5*) and demonstrate that current vaccination efforts are resulting in substantial preventive benefits among working-age adults. They reinforce CDC’s recommendation of full 2-dose immunization with mRNA vaccines. COVID-19 vaccination is recommended for all eligible persons, which currently varies by location in the United States.

**Summary**

**What is already known about this topic?**

Messenger RNA (mRNA) COVID-19 vaccines have been shown to be effective in preventing symptomatic SARS-CoV-2 infection in randomized placebo-controlled Phase III trials.

**What is added by this report?**

Prospective cohorts of 3,950 health care personnel, first responders, and other essential and frontline workers completed weekly SARS-CoV-2 testing for 13 consecutive weeks. Under real-world conditions, mRNA vaccine effectiveness of full immunization (≥14 days after second dose) was 90% against SARS-CoV-2 infections regardless of symptom status; vaccine effectiveness of partial immunization (≥14 days after first dose but before second dose) was 80%.

**What are the implications for public health practice?**

Authorized mRNA COVID-19 vaccines are effective for preventing SARS-CoV-2 infection in real-world conditions. COVID-19 vaccination is recommended for all eligible persons.

## Footnotes

* Occupational categories: primary health care personnel (physicians, physician assistants, nurse practitioners, and dentists), other allied health care personnel (nurses, therapists, technicians, medical assistants, orderlies, and all other persons providing clinical support in inpatient or outpatient settings), first responders (firefighters, law enforcement, corrections, and emergency medical technicians), other essential and frontline workers (workers in hospitality, delivery, and retail; teachers; and all other occupations that require contact within 3 feet of the public, customers, or coworkers as a routine part of their job).

† An additional five participants received the Janssen COVID-19 vaccine (Johnson & Johnson), resulting in 2,961 vaccinated participants.

§ Person-days is an estimate of the time-at-risk (to SARS-CoV-2 infection) that each participant contributed to the study.

¶ Arizona Healthcare, Emergency Response and Other Essential Workers Surveillance Study (HEROES); Research on the Epidemiology of SARS-CoV-2 in Essential Response Personnel (RECOVER).

**
https://preprints.jmir.org/preprint/28925

†† 45 C.F.R. part 46; 21 C.F.R. part 56; 42 U.S.C. Sect. 241(d), 5 U.S.C. Sect. 552a, 44 U.S.C. Sect. 3501 et seq.
